# A Hospital Meeting at Downing Street

**Published:** 1918-08-03

**Authors:** 


### A Hospital Meeting at Downing Street.

### An Experimental Limb Shop.

THE Council of the Prince of Wales' Hospital for Limbless Sailors and Soldiers, Cardiff, held its half-yearly meeting at 10 Downing Street, on July 25, by kind permission of Mrs. Lloyd George. This is the second occasion for this hospital to hold its Council meeting in the house of the Prime Minister. The attendance included Mrs. Lloyd George, in the chair. Lord Aberdare; Surgeon-General Sir W. H. Norman, K.C.B., R.N.; Colonel J. Lynn Thomas, C.B., C.M.G., and Mrs. Thomas; Mr. and Mrs. W. Percy Miles; and the Secretary, Mr. Gilbert D. Shepherd, F.C.A. The report of the Executive Committee showed that from the opening of the hospital on May 7, 1917, to June 30, 1918 (approximately fourteen months), 509 patients were fitted with artificial limbs (363 legs and 146 arms), including 21 double amputation cases. The whole of these limbs had been made at the hospital in the limb shops and, in addition, 188 pensioners (previously fitted at Roehampton or Cardiff) had been received for repairs or re-fitting.

EXPERIMENTAL LIMB SHOP.

A grant of £250 was made by the Treasury on June 18 for a special workshop in which experiments for improving artificial limbs might be carried out under the direct supervision of the consulting surgeons of the hospital. H.R.H. the Prince of Wales has sent £100 for the same purpose.

ENDOWMENT FUND.

Further gifts to the endowment fund were announced from Lord and Lady Aberdare, £1,050; Lord and Lady Glanusk, £1,050; Sir William James and Lady Thomas, £1,050; also a promise of £1,050 made the previous day by Councillor J. C. Gould, all of these being for the endowment of beds. The endowment fund at present amounts to £32,500, and all of it has been invested in National War Bonds. The vacancy on the Council caused by the death of Mr. J. P. Cadogan has been filled by the appointment of Lt.-Col. John A. Jones. Mr. W. Percy Miles has been appointed chairman of the Executive Committee. It was arranged to hold a special meeting of the Council in Cardiff on October 24, when Mrs. Lloyd George promised to attend. The minutes recorded the good work of Miss Clara Deacon, the matron and commandant, and of Mr. Gilbert Shepherd, the secretary.

